# Minimizing Incision in Living Donor Liver Transplantation: Initial Experience and Comparative Analysis of Upper Midline Incision in 115 Recipients

**DOI:** 10.3389/ti.2024.12536

**Published:** 2024-05-21

**Authors:** Amit Rastogi, Ankur A. Gupta, Raghav Bansal, Fysal Kollanta Valappil, Kamal S. Yadav, Suchet Chaudhary, Prashant Bhangui, Swapnil Dhampalvar, Narendra S. Choudhary, Neeraj Saraf, Arvinder S. Soin

**Affiliations:** ^1^ Institute of Liver Transplantation, Medanta, Gurugram, India; ^2^ Hepatology, Institute of Liver Transplantation and Regenerative Medicine, Medanta, Gurugram, India

**Keywords:** living donor liver transplantation, recipient surgery, upper midline incision, wound complications, incision scar

## Abstract

Living donor liver transplantation (LDLT) needs “Mercedes Benz” or “J-shaped” incision, causing short and long-term complications. An upper midline incision (UMI) is less invasive alternative but technically challenging. Reporting UMI for recipients in LDLT vs. conventional J-shaped incision. Retrospective analysis, July 2021 to December 2022. Peri-operative details and post-transplant outcomes of 115 consecutive adult LDLT recipients transplanted with UMI compared with 140 recipients with J-shaped incision. Cohorts had similar preoperative and intraoperative variables. The UMI group had significant shorter time to ambulation (3 ± 1.6 vs. 3.6 ± 1.3 days, *p* = 0.001), ICU stay (3.8 ± 1.3 vs. 4.4 ± 1.5 days, *p* = 0.001), but a similar hospital stay (15.6±7.6 vs. 16.1±10.9 days, *p* = 0.677), lower incidence of pleural effusion (11.3% vs. 27.1% *p* = 0.002), and post-operative ileus (1.7% vs. 9.3% *p* = 0.011). The rates of graft dysfunction (4.3% vs. 8.5% *p* = 0.412), biliary complications (6.1% vs. 12.1% *p* = 0.099), 90-day mortality (7.8% vs. 12.1% *p* = 0.598) were similar. UMI-LDLT afforded benefits such as reduced pleuropulmonary complications, better early post-operative recovery and reduction in scar-related complaints in the medium-term. This is a safe, non-inferior and reproducible technique for LDLT.

## Graphical Abstract

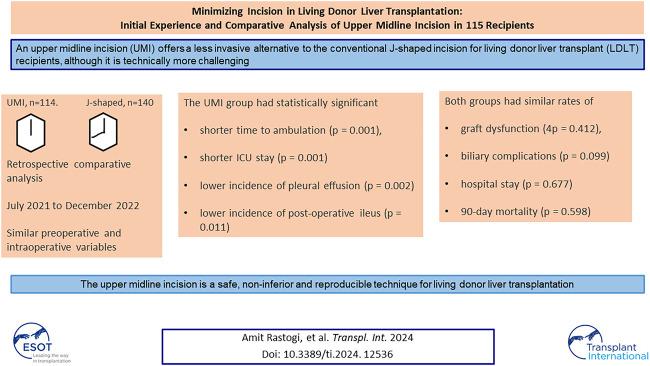

## Introduction

The incision used for liver recipient surgery has evolved over the years from the classic “Mercedes Benz” to the “J shaped” or “Hockey stick” incision [1, 2]. Both incisions can provide sufficient exposure, but they involve extensive cutting of the abdominal muscles, which can pose short-term concerns such as pain, hematoma, poor respiratory compliance, wound infection, dehiscence, and paresthesia over the scar. Long-term complications may include scar formation, hernia, and loss of sensation in the upper abdomen [1].

The midline incision, on the other hand, offers excellent exposure to the surgical field while avoiding muscle cutting [2]. It passes through the avascular rectus sheath, causing minimal damage to the subcutaneous nerves and blood vessels. However, surgeons have often avoided using smaller incisions in recipients due to the risk of bleeding associated with portal hypertension during hepatectomy and the technical challenges of achieving perfect vascular anastomoses with a short warm ischemia time during graft implantation.

After the initial reports of successful utilization of an upper midline incision [2] or laparoscopic assistance with such an incision (hybrid procedure) for recipient surgery [3, 4], Jochmans et al reported the feasibility of a single xipho-pubic laparotomy for hepatectomy, nephrectomy, and transplantation in cases of polycystic disease for simultaneous liver-kidney transplant [5] while Fonseca-Neto et al reported recipient surgery with whole liver cadaveric donor grafts through an upper midline incision [6]. This hybrid procedure continues to be published in the current literature [7–10] and has now been reported in a pediatric recipient [11].

Based on our extensive experience with the use of the midline incision for liver donor surgery, we introduced an upper midline abdominal incision for recipient hepatectomy and liver graft implantation in LDLTs and modified our surgical steps as described. It is noteworthy that the published literature on this topic is so far based on a small number of patients. In this report, we aim to contribute our experience with 115 consecutive cases of recipient surgery performed with an upper midline incision which, to the best of our knowledge, represents the largest reported experience with this technique. The aim of this study was to compare the recipient outcomes of a midline incision versus a conventional “J-shaped” incision in LDLT.

## Materials and Methods

This was a retrospective analysis of a prospectively maintained, comprehensive database of all liver transplants performed at our center. A total of 115 adult recipients underwent LDLT via a midline incision between July 2021 and December 2022. Peri-operative details and post-transplant outcomes of this group were analyzed and compared with those of a group of 140 recipients who underwent LDLT via a J-shaped incision during the same period. The patients were randomly selected to receive either type of incision. The surgical team remained the same in both groups.

This study was approved by the Institutional Review Board of the Hospital.

### Selection of Midline Recipients

Pediatric, dual-lobe, re-transplant, and combined liver-kidney transplant recipients were excluded. In the initial part of our experience with the first five midline LDLTs, recipients with a high body mass index (BMI) greater than 35, a history of previous abdominal surgery, or a history of spontaneous bacterial peritonitis (SBP) were also excluded. All excluded cases were not part of the present comparative analysis. Subsequently, all patients were randomized to either group.

Two different incisions were used for donor surgery in the midline cohort. Open donor hepatectomy was performed in 91 cases using the upper midline incision, while 24 cases underwent a total robotic donor hepatectomy. For the “J incision” cohort 121 donors underwent open donor hepatectomy (conventional and midline) while 19 donors underwent robotic donor hepatectomy.

### Surgical Technique

**FIGURE 1:**
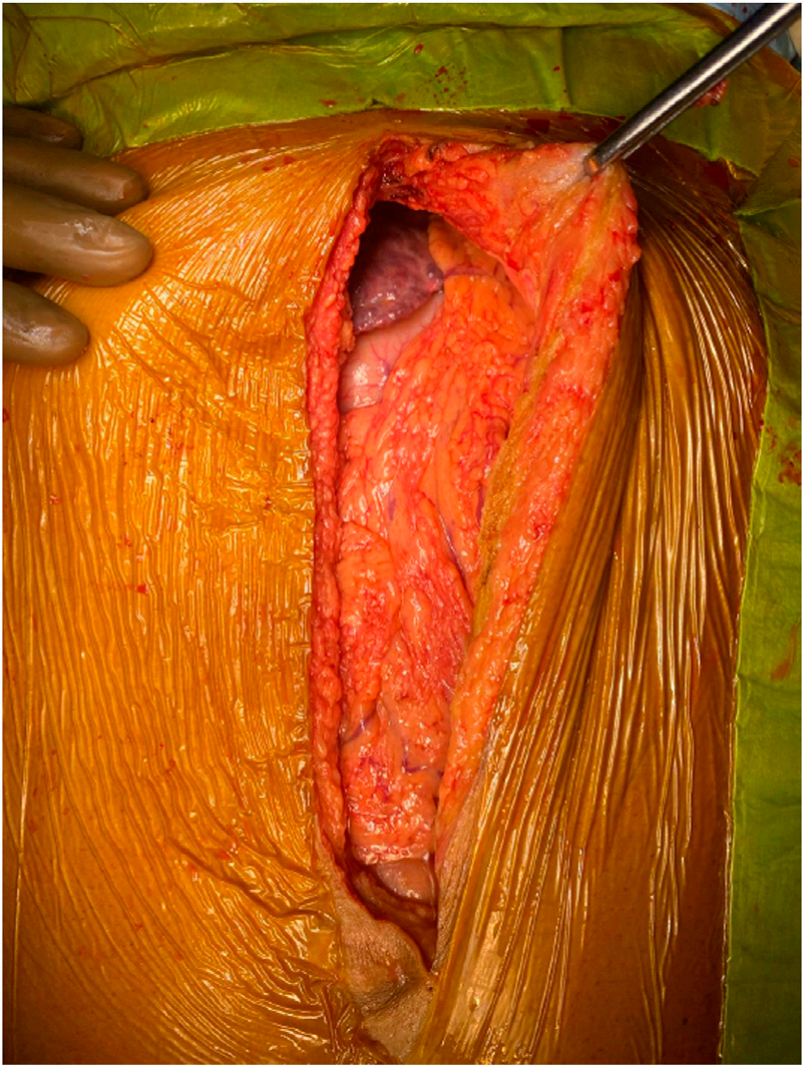
Midline skin incision.

**FIGURE 2:**
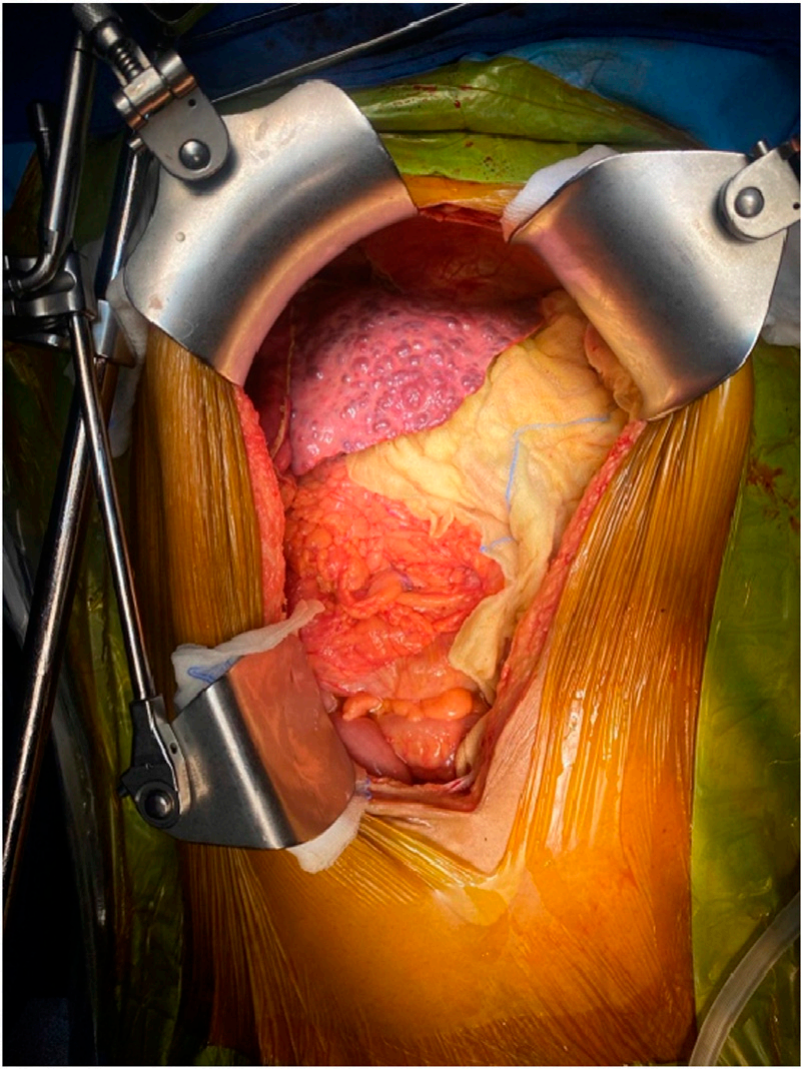
Placement of abdominal retractor blades: two costal margin retractors and one right abdominal wall retractor.

The upper midline incision extended from the xiphoid to the umbilicus and curved around it if needed (Figure 1). To achieve a wide elliptical exposure, we used Thomson’s Retractor™ with conventional bilateral costal retractors. During the hepatectomy, right lateral traction was applied to the right abdominal wall at the lower edge of the incision using a side arm attachment of the Thomson’s Retractor™ (Figure 2) and later on the left side during implantation. This maneuver increased the space around the porta, and the stomach and colon/bowel were packed down with surgical sponges.

**FIGURE 3:**
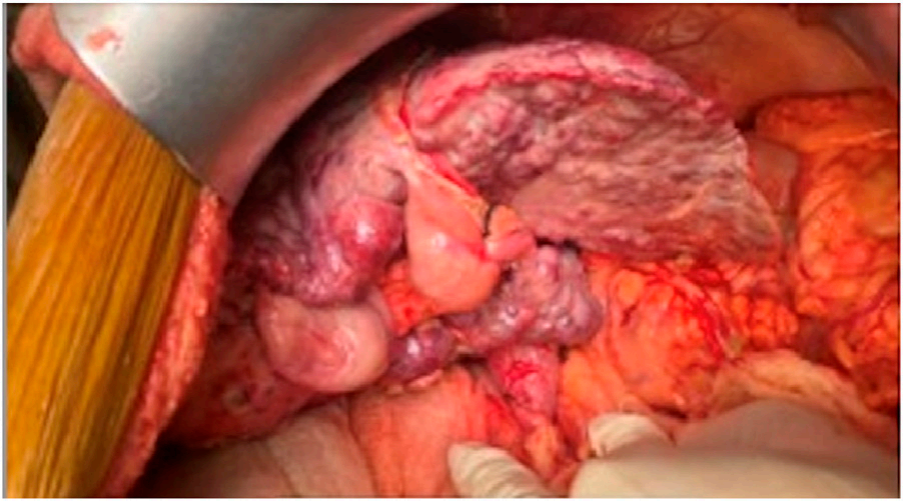
Completion of portal dissection: native liver in the anhepatic phase.

**FIGURE 4:**
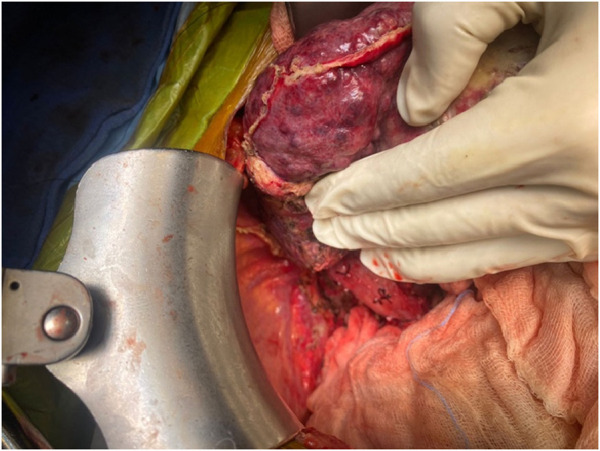
Right lobe mobilization.

**FIGURE 5:**
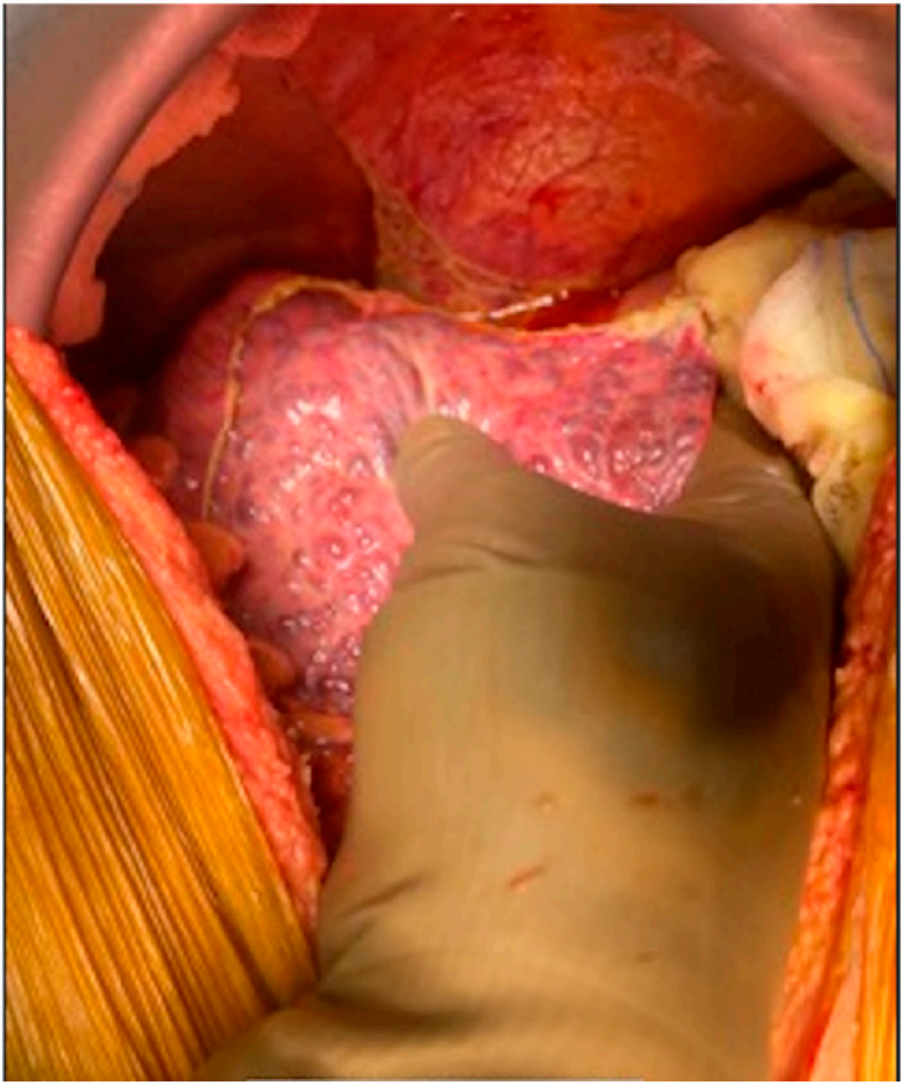
Left lobe mobilization.

**FIGURE 6:**
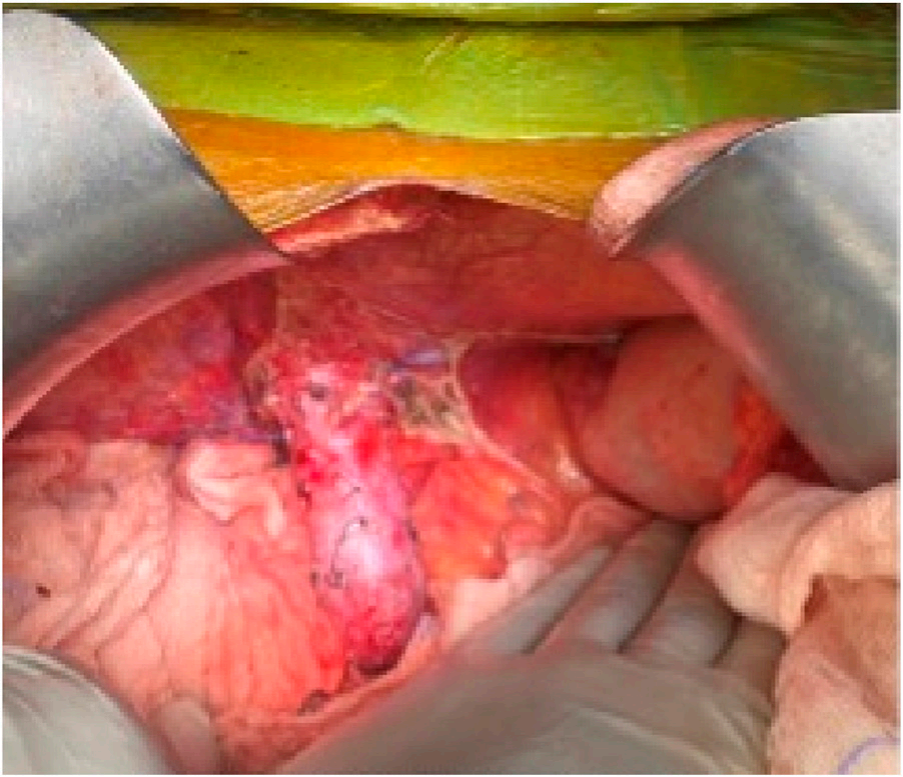
Abdominal cavity after removal of native liver.

The salient difference from the conventional technique is the early portal dissection and division of the hepatic arteries and bile ducts. If portal hypertension is severe, the portal vein is also divided before right lobe mobilization. This helps to reduce both, the blood loss and the size of the liver for subsequent mobilization of the right lobe (Figure 3). This is performed from the inferior to the superior aspect of the liver instead of the conventional lateral to medial mobilization. With increasing experience, we have been able to avoid the division of the portal vein prior to the mobilization of the right lobe in more than 50% of our recipients. Right lobe mobilization was followed by left lobe mobilization (Figures 4, 5), posterior and anterior IVC dissection, ligation of the hepatic veins, and removal of the diseased liver (Figure 6).

Bench surgery was performed in the usual manner with respect to the anatomy of the graft. In the majority of recipients, we performed a “plasty” of the end of the MHV extension with the RHV orifice to allow for a single RHV and MHV outflow anastomosis.

**FIGURE 7:**
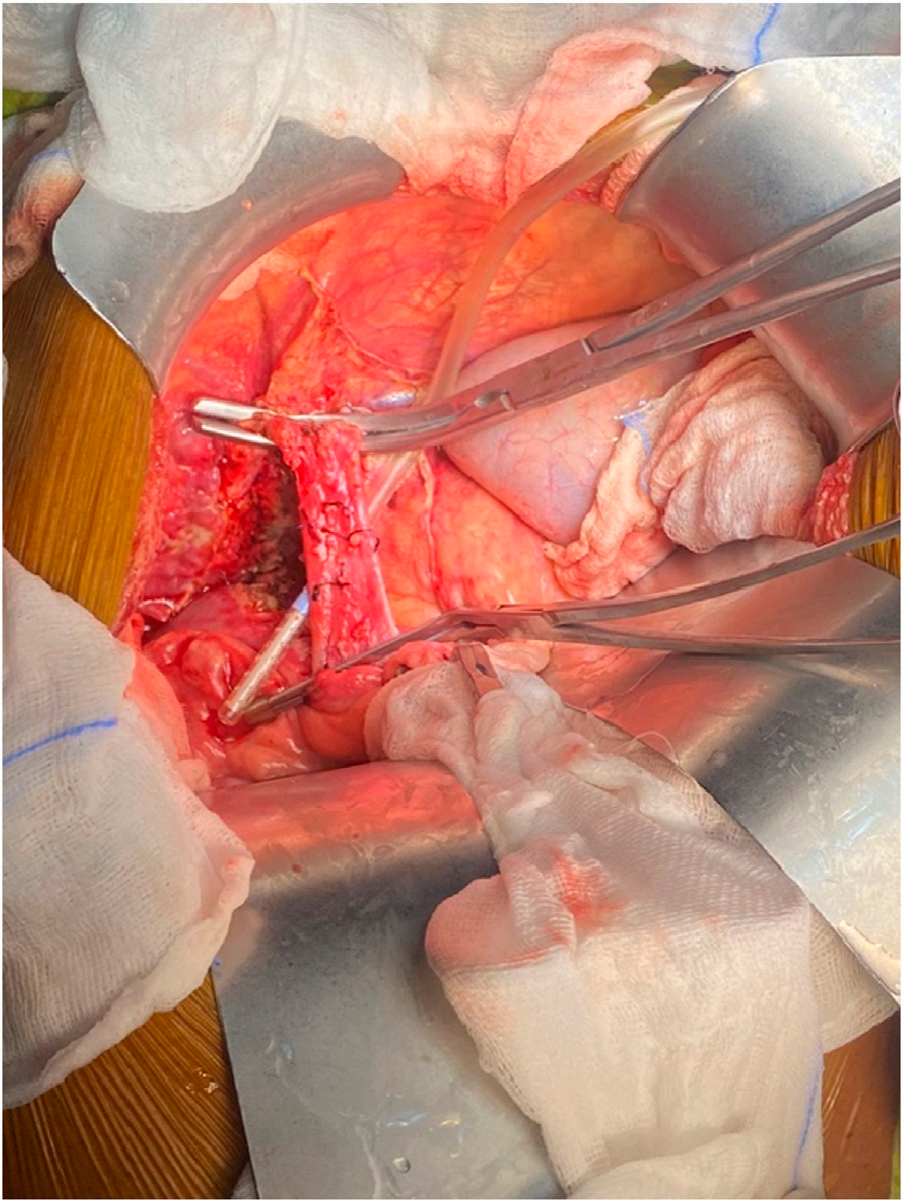
IVC cross-clamping.

**FIGURE 8:**
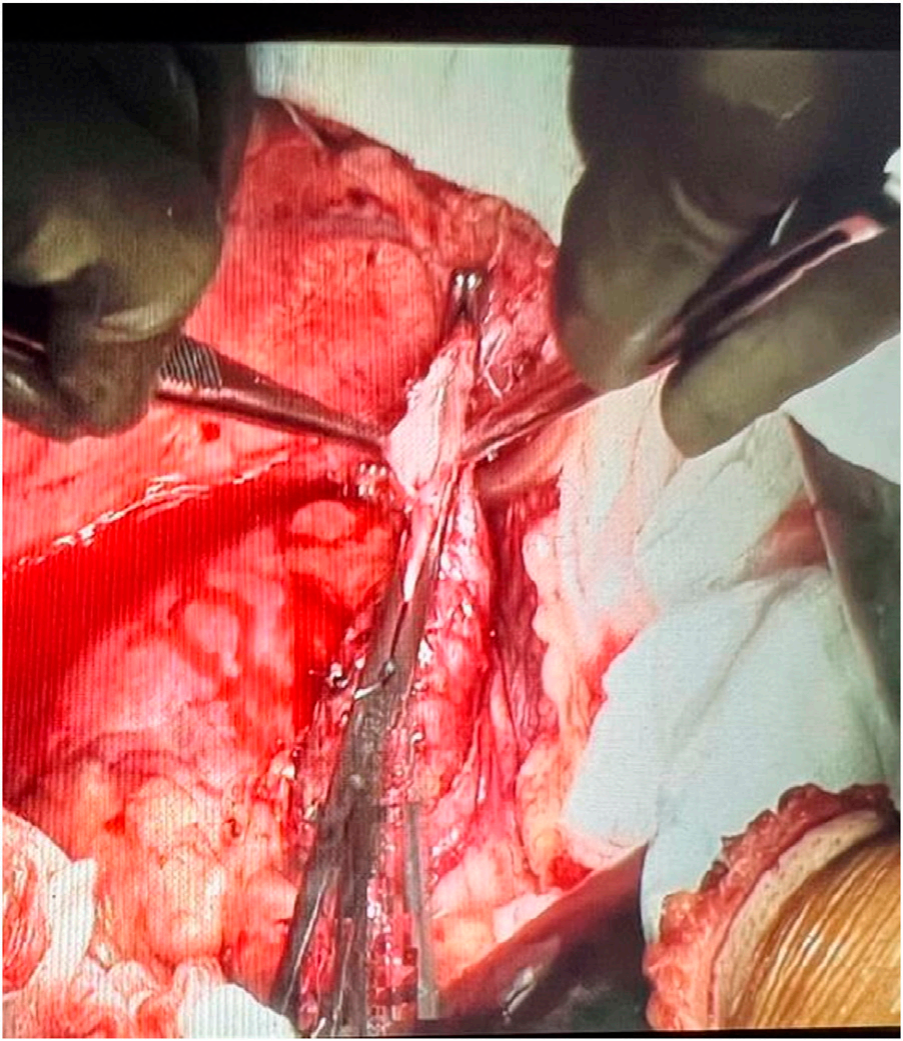
IVC side clamping.

Graft implantation was done by cross-clamping (or side-clamping in cases of renal or cardiac dysfunction) the IVC (Figures 7, 8). The supra-hepatic caval clamp remained the same (Ulrich Swiss™ IVC clamp 280 mm) as in the conventional incision. However, to clamp the lower IVC, a longer clamp (Debakey renal artery clamp) was used from the left side (versus the right side in the conventional technique). Longer clamps were also used for side caval clamping (FB508R Debakey-Satinsky Clamp, Aesculap US).

**FIGURE 9:**
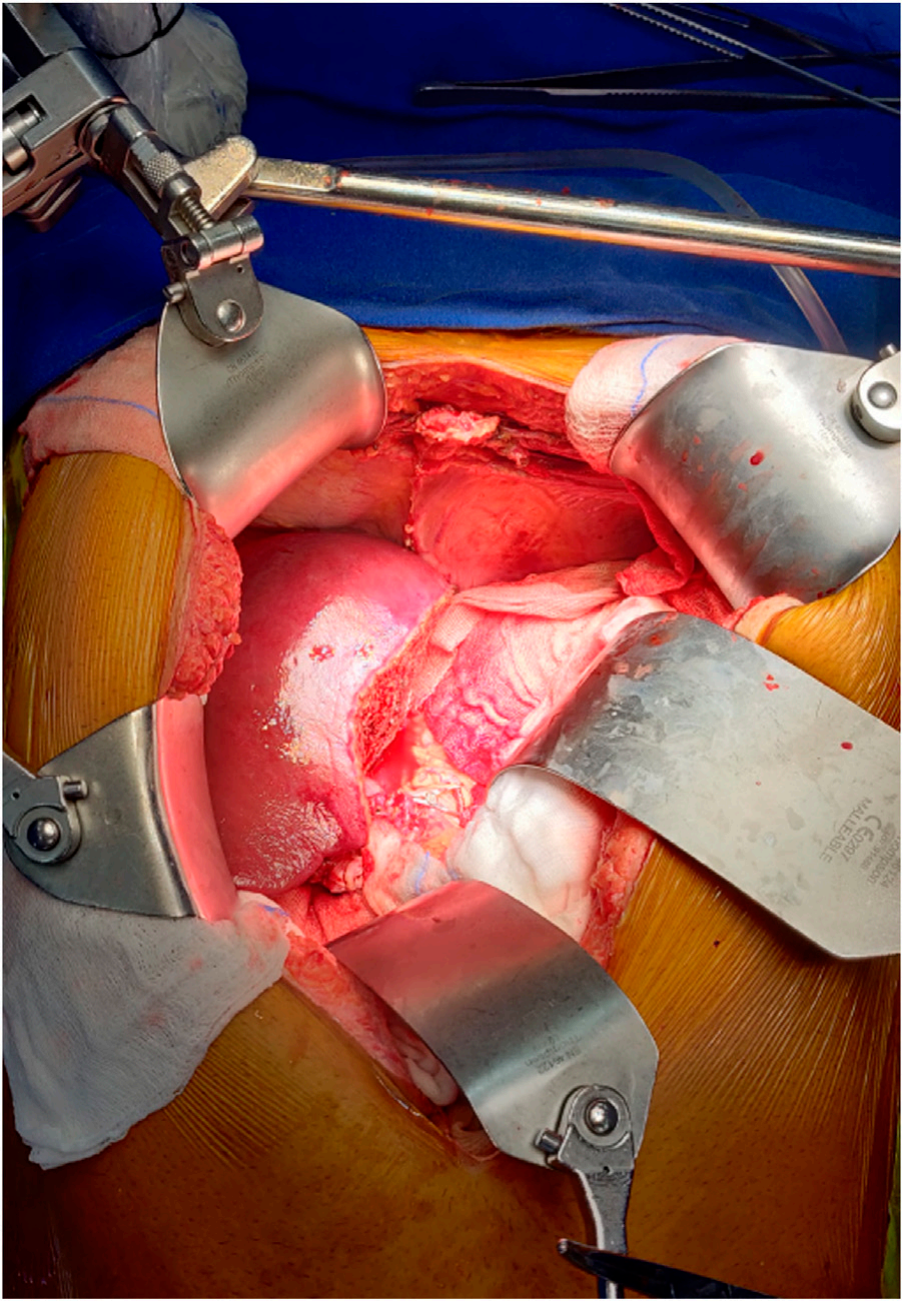
Implanted graft.

Implantation of the outflow veins (RHV, MHV, and inferior hepatic veins) and portal vein was followed by graft reperfusion and subsequent hepatic artery and bile duct anastomoses (Figure 9).

The bench reconstruction, implantation technique, and postoperative management protocols for all recipients were the same for all recipients irrespective of the incision used. Recipients were nursed in the ICU for 2–4 days and then transferred to the ward.

### Statistical Analysis

The analysis involved the profiling of patients based on various demographic, clinical, and laboratory parameters. Descriptive statistics were used to analyze quantitative variables, which were reported as means and standard deviations. Categorical variables were expressed as absolute numbers and percentages. The independent Student’s t-test was used to compare the means between independent groups. Cross tables were generated, and the Chi-square test was used to test for associations. A *p*-value of < 0.05 was considered statistically significant. All statistical analyses were performed using SPSS software, version 24.0.

## Results

The overall and mean follow-up periods for both groups were 6–19 months (mean 8.2 months ± 6.5). Tables 1–3 show the preoperative and intraoperative characteristics, and postoperative outcomes of the two groups of recipients.

**TABLE 1:** Pre-operative characteristics of recipients in the midline and conventional incision groups.

Pre-operative variables	Midline incision (*n* = 115)	J shaped incision (*n* = 140)	*p*-value
Men (no.)	95 (82.6%)	111 (79.3%)	0.503
Age (years)	49.8 ± 11.6	48 ± 13.5	0.246
BMI (Kg/m^2^)	24.7 ± 4.2	24.3 ± 5.1	0.571
Moderate to gross ascites	83 (72.2%)	99 (71.0%)	0.833
Portal vein thrombosis (Yerdel grade 2 or more)	3 (2.6%)	6 (4.3%)	0.464
CTP score	9.1 ± 2.2	8.7 ± 2.2	0.114
Comorbidities			
Diabetes mellitus	46 (40%)	42 (30%)	0.095
CAD	4 (3.5%)	2 (1.4%)	0.283
HCC	23 (20%)	21 (15%)	0.293
Etiology of Liver Disease			
HBV	10 (8.7%)	14 (10%)	0.723
HCV	14 (12.2%)	17 (12.1%)	0.994
ALD	31 (27%)	33 (23.6%)	0.535
Autoimmune	9 (7.8%)	6 (4.3%)	0.232
NASH	18 (15.7%)	15 (10.7%)	0.242
ATT induced	0 (0%)	1 (0.7%)	0.364
HEV	0 (0%)	2 (1.4%)	0.198
Wilson’s Disease	3 (2.6%)	2 (1.4%)	0.499
Cryptogenic	12 (10.4%)	24 (17.1%)	0.126

ALD, alcoholic liver disease; ATT, anti-tubercular therapy; CAD, coronary artery disease; CTP, Child-Turcotte-Pugh score; HCC, hepatocellular carcinoma; HBV, Hepatitis B virus; HCV, Hepatitis C virus; HEV, Hepatitis E virus; MELD, Model for End-Stage Liver Disease score; NASH, Non-alcoholic steatohepatitis.

**TABLE 2:** Categorization and comparison of MELD score of recipients in the midline and conventional incision groups.

MELD	Midline incision (*n* = 115)	J shaped incision (*n* = 140)
<21	88 (76.5%)	96 (68.6%)
21–30	25 (21.7%)	37 (26.4%)
>30	2 (1.7%)	7 (5.0%)

Chi-Square Value = 3.026, *p*-value = 0.220.

**TABLE 3:** Intra-operative characteristics of recipients in the midline and conventional incision groups.

Intraoperative variables	Midline incision (*n* = 115)	J shaped incision (*n* = 140)	*p*-value
Operative Time (minutes)	749.5 ± 248.1	701.4 ± 165.7	0.069
Donors undergoing robotic hepatectomy	24 (20.9%)	18 (12.9%)	0.088
Right-lobe grafts	113 (98.2%)	134 (95.7%)	0.246
Graft weight (grams)	677.9 ± 129.3	652.3 ± 133.1	0.125
GRWR	1.02 ± 0.23	1.0 ± 0.26	0.461
RL recipients with GRWR <0.8 (%)	16 (13.9%)	23 (16.4%)	0.657
>1 graft hepatic duct	65 (56.5%)	68 (48.5%)	0.548
IVC Clamp Time (minutes)	39.9 ± 12.4	42.3 ± 9.8	0.087
Partial/side clamping	63 (54.8%)	73 (52.1%)	0.633
CIT (minutes)	101.2 ± 39.1	112.5 ± 36.8	0.018*
WIT (minutes)	29.7 ± 10.6	31 ± 8.6	0.298
PRBC transfusion (units)	6.3 ± 4.3	5.3 ± 4.1	0.058
Blood loss (mL)	2241.3 ± 1253.8	2101.4 ± 1106.9	0.345
Blood lactate prior to transfer to the ICU (mmol/L)	4.92 ± 2.59	5.2 ± 3.27	0.46

CIT, cold ischemia time; GRWR, graft-to-recipient weight ratio; ICU, intensive care unit; IVC, inferior vena cava; PRBC, packed red blood cells; RL, right lobe; WIT, warm ischemia time. * indicate significant *p* value, < 0.05.

The midline incision and J-shaped incision groups had similar preoperative variables and demographic characteristics, as there were no significant differences in age, gender, BMI, CTP score, MELD score, hepatocellular carcinoma, or underlying etiology (Table 2). In addition, the prevalence of diabetes mellitus, and CAD was not significantly different between the two groups.

As shown in Table 3, there were no statistically significant differences between the midline incision and J-shaped incision cohorts in terms of operative time, frequency of right lobe grafts, open versus robotic donor hepatectomy, graft weight, graft-to-recipient weight ratio (GRWR), the proportion of patients with low GRWR grafts (<0.8%), number of graft bile ducts, IVC clamping time, the proportion with partial versus total IVC clamping during implantation, warm ischemia time (WIT), blood loss, transfusion requirement, and blood lactate prior to transfer to the ICU. However, cold ischemia time (CIT) was shorter in the midline incision group than in the J-shaped incision group (values 101.2 ± 39.1 vs. 112.5 ± 36.8; *p* = 0.018).

Table 4 shows the post-operative parameters and outcomes between the two groups of recipients. There was no statistically significant difference in blood lactate on the first post-operative day or the duration of the requirement for mechanical ventilation between the two groups. However, the midline incision group had a statistically significant shorter time to ambulation (*p* = 0.001), a shorter ICU stay (*p* = 0.001), but a similar hospital stay compared to the J-shaped incision group. At the same time, the J-shaped incision cohort had significantly higher rates of pleural effusion, transfusion requirements, and post-operative ileus. While the incidence of wound-related complications such as seroma, wound infection, and dehiscence was higher in the J-shaped incision group, the difference was not statistically significant. The rates of graft dysfunction, re-exploration rate for bleeding, biliary complications, and mortality were similar between the two groups.

**TABLE 4:** Post-operative outcomes of recipients in the midline and conventional incision groups.

Post-operative variables	Midline incision (*n* = 115)	J shaped incision (*n* = 140)	*p*-value
Blood lactate on the first postoperative day (mmol/L)	3.18 ± 2.05	3.79 ± 2.76	0.056
Mechanical Ventilation (days)	1.4 ± 1.1	1.3 ± 0.9	0.188
Time to ambulation (days)	3.0 ± 1.6	3.6 ± 1.3	0.001*
ICU stay (days)	3.8 ± 1.3	4.4 ± 1.5	0.001*
Hospital stay (days)	15.6 ± 7.6	16.1 ± 10.9	0.677
Wound-related complications	12 (10.4%)	18 (12.9%)	0.55
Graft dysfunction	5 (4.3%)	12 (8.5%)	0.412
Biliary complications	7 (6.1%)	17 (12.1%)	0.099
Pleural effusion	13 (11.3%)	38 (27.1%)	0.002*
Transfusion requirement	10 (8.7%)	25 (17.9%)	0.034*
Re-exploration rate for bleeding	3 (2.6%)	2 (1.4%)	0.245
Post-operative ileus	2 (1.7%)	13 (9.3%)	0.011*
Mortality (90 days)	9 (7.8%)	17 (12.1%)	0.598
Incisional hernia	5 (4.3%)	5 (3.6%)	0.774

ICU, intensive care unit. * indicate significant *p* value, < 0.05.

In the midline incision cohort, four patients required an extension of the incision, and three needed a muscle-cutting (conventional) incision. These conversions were done in the early part of the experience, and all three were converted after liver explantation. Two of these patients developed bowel edema after the anhepatic phase, and one patient experienced bleeding from the RHV anastomosis just before abdominal closure. A fourth patient required an extension of the midline incision to below the umbilicus due to a thick muscular wall that reduced the working space. With increasing experience, we felt that this extension could potentially mitigate the need for conversion to a muscle incision. Adequate exposure was maintained, and muscle cutting was avoided in these cases.

## Discussion

Multiple authors have documented the safety of using the upper midline incision in major hepatectomies, including those in liver donors [12–16] and more recently in patients with chronic liver disease and liver fibrosis [17]. Our team has also incorporated the use of the midline incision in liver recipients, capitalizing on our experience with its application in donors and the recognized benefits of this incision over those that require cutting through muscle tissue. We have successfully performed over 500 donor hepatectomies using the midline incision. In our cohort of midline incision recipients, 91 donor surgeries were done with an open upper midline approach while 24 donors underwent robotic hepatectomy.

We opted for the pure open upper midline incision approach for liver surgery over the totally minimally invasive [8] or laparoscopically assisted midline approaches [3, 4, 9] for several reasons. First, the complete laparoscopic or robotic approach is not suitable for many recipients due to their range of conditions, portal hypertension, technical difficulties in vascular and biliary anastomoses, and graft anatomical variations encountered. Second, the anastomoses for graft implantation are in the plane of the IVC and the hepato-duodenal ligament, which are easily accessible through a midline incision. Third, the use of good retraction, long instruments, and modification of the surgical technique enables easy standardization of the operative steps for use by all surgeons on the team, rather than restricting it only to those with expertise in minimally invasive surgery. We believe that a pure open upper midline laparotomy procedure is also safer than a hybrid approach with its natural benefits in postoperative rehabilitation [3].

The upper midline incision can provide adequate exposure for recipient surgery. Midline incisions allow for easy left lobe mobilization and access to the suprahepatic vena cava and provide good exposure for both the hepatic and portal vein anastomoses [2]. This approach has been safely used in LT recipients receiving whole grafts from deceased donors [6]. Additionally, a midline incision extending from the xiphoid process to the pubis has been reported to be adequate for hepatectomy, native nephrectomy, and simultaneous liver-kidney (SLK) transplantation in patients with polycystic disease [5]. More recently, the upper midline incision was reported to be adequate for graft implantation in a pediatric patient, further highlighting its usefulness in the LT setting [11].

In the initial phase of the study, the exclusion criteria were outlined as previously described. After the first five cases, the team consistently utilized an upper midline incision for all subsequent cases, regardless of recipient characteristics like BMI, height, etc., which may have suggested a limited working field [6]. The incision was extended as needed based on the situation keeping patient safety as our primary objective. As discussed above, with increasing experience, we have not resorted to extending to a muscle-cutting incision and extending along the midline below the umbilicus in occasional patients.

A learning curve for performing donor hepatectomy through an upper midline incision has been reported in the literature [18]. In our group, the initial cases of upper midline recipient surgery were performed by senior surgeons with extensive experience, as recommended in previous studies [2]. As experience was gained, other surgeons within the group also began to perform the procedure.

The amount of blood loss in the two cohorts of patients was found to be comparable in the study, which is consistent with previously published experience [3]. There was no notable distinction between the two groups in terms of immediate post-operative lactate levels before transfer to the ICU, suggesting that both groups exhibited comparable metabolic responses during surgery. CIT is multifactorial and a small difference was observed between the two cohorts in our study, the significance of which remains inconclusive.

The advantages of a midline incision in comparison to transverse incisions are that it preserves the innervation and avoids muscle disruption, resulting in less postoperative pain [6]. Patients who undergo midline incisions have reported better results in terms of numbness and cutaneous sensation [19, 20]. Midline incisions also offer the benefit of a decreased risk of wound complications, such as infection and dehiscence, in contrast to utilizing a transverse incision [6, 11]. Avoidance of abdominal muscle and nerve disruption also leads to reduced use of analgesics and early ambulation and rehabilitation [19]. Patients also exhibit greater compliance with physiotherapeutic maneuvers such as spirometry, leading to a shorter ICU stay [3, 6, 11]. Our patients in the midline incision group showed a comparable trend, with significantly shorter time to ambulation, a lower incidence of postoperative pleural effusion and ileus, and a shorter ICU stay.

**FIGURE 10:**
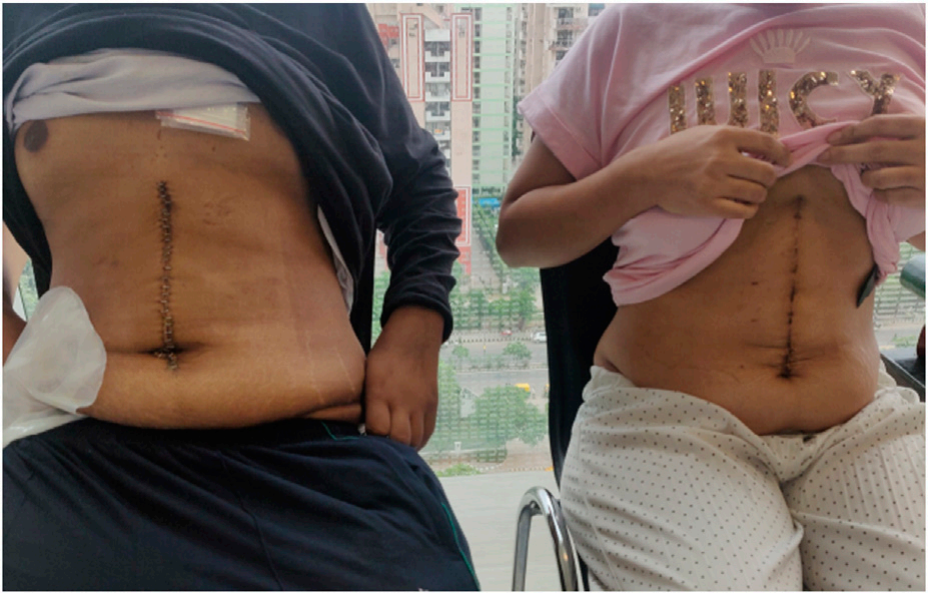
First case, July 2021: recipient on the left and donor on the right.

In previous donor studies, a midline incision was found to offer better cosmesis and increased self-confidence, with patients reporting good self-assessment of appearance and daily activities [19, 20]. The majority of our patients have expressed satisfaction with the incision at follow-up clinics (Figure 10). However, a formal questionnaire-based analysis has yet to be performed.

Earlier studies have suggested that incisional hernia occurrence after liver transplant is higher in cases with an element of midline incision compared to those without [21, 22]. However, a recent meta-analysis of incisional hernia formation in hepatobiliary surgery found no significant difference in incisional hernia formation between the hybrid (with midline incision) and transverse incision groups [23]. Another recent meta-analysis reported a median incidence of incisional hernia of 15.1%, with a median time of 42.9 months post-liver transplantation [24]. As our study focuses on the initial experience with the midline incision, our follow-up period is relatively short. Five patients in each group have developed incisional hernia so far, but none of them have undergone surgery yet. Hence, comparing the incidence of incisional hernia between the two cohorts may not be meaningful at this stage.

The limitations of the current study include the lack of a randomized controlled trial design and its retrospective nature. Another limitation is the relatively short follow-up period, which precludes an adequate assessment of complications such as incisional hernia. Finally, no objective assessment of patient satisfaction was conducted, which could be addressed through a questionnaire-based study.

To the best of our knowledge, this is the largest series to date, but the sample size could still be considered relatively small, which may limit the generalizability of the results. Furthermore, the study was conducted at a single center, which may limit the external validity of the findings to other centers with different patient populations and surgical teams.

## Conclusion

Our initial experience with midline LDLT has yielded promising results, with favorable outcomes for the recipients. We have demonstrated that a completely open midline approach is possible without requiring the mobilization of the right lobe using laparoscopic or robotic techniques. With increasing experience, we believe that this approach can be extended to most patients undergoing LDLT.

Our midline incision technique offers a safe, non-inferior, and reproducible procedure with potential benefits such as reduced pleuropulmonary complications and better early post-operative recovery, due to the non-muscle-cutting nature of the incision. We believe that the reduction in incision size and the resulting scar may lead to better acceptance of liver transplant surgery. The continued use of muscle-cutting incisions in recipient surgery is due to the technical complexity involved. Nevertheless, more prospective data are needed to verify these initial findings.

## Data Availability

The raw data supporting the conclusion of this article will be made available by the authors, without undue reservation.

## Abbreviations

ALD, Alcoholic liver disease; ATT, Anti-tubercular therapy; BMI, Body Mass Index; CAD, Coronary artery disease; CIT, Cold ischemia time; CTP, Child-Turcotte-Pugh score; GRWR, Graft-to-recipient weight ratio; HbcAb, Hepatitis B core antibody; HCC, Hepatocellular carcinoma; HBV, Hepatitis B virus; HCV, Hepatitis C virus; HEV, Hepatitis E virus; ICU, Intensive care unit; IV, Intravenous; IVC, Inferior vena cava; LDLT, Living donor liver transplantation; LT, Liver transplantation; MELD, Model for End-Stage Liver Disease score; MHV, Middle hepatic vein; n, number; NASH, Non-alcoholic steatohepatitis; PRBC, Packed red blood cells; RHV, Right hepatic vein; RL, Right lobe; SBP, Spontaneous bacterial peritonitis; SLK, Simultaneous liver-kidney; SPSS, Statistical Package for the Social Sciences; WIT, Warm ischemia time.
